# Enhanced trypsin thermostability in *Pichia pastoris* through truncating the flexible region

**DOI:** 10.1186/s12934-018-1012-x

**Published:** 2018-10-25

**Authors:** Lin Liu, Haoran Yu, Kun Du, Zhiyan Wang, Yiru Gan, He Huang

**Affiliations:** 1 Department of Biochemical Engineering, School of Chemical Engineering and Technology, Tianjin University, Tianjin, 300350 China; 2 Key Laboratory of System Bioengineering, Ministry of Education, Tianjin University, Tianjin, 300350 China; 3 Collaborative Innovation Center of Chemical Science and Engineering, Tianjin, 300350 China; 4 Present Address: Department of Biochemical Engineering, University College London, Gordon Street, London, WC1H 0AH UK

**Keywords:** Thermostability, Trypsin, Flexible region, Truncation, Ω-loop

## Abstract

**Background:**

High thermostability is required for trypsin to have wider industrial applications. Target mutagenesis at flexible regions has been proved to be an efficient protein engineering method to enhance the protein thermostability.

**Results:**

The flexible regions in porcine trypsin were predicted using the methods including molecular dynamic simulation, FlexPred, and FoldUnfold. The amino acids 78–90 was predicted to be the highly flexible region simultaneously by the three methods and hence selected to be the mutation target. We constructed five variants (D3, D5, D7, D9, and D11) by truncating the region. And the variant D9 showed higher thermostability, with a 5 °C increase in *T*_opt_, 5.8 °C rise in $T_{50}^{10}$, and a 4.5 °C rise in *T*_m_, compared to the wild-type. Moreover, the half-life value of the variant D9 was also found to be dramatically improved by 46 min. Circular dichroism and intrinsic fluorescence indicated that the structures had no significant change between the variant D9 and the wild-type. The surface hydrophobicity of D9 was measured to be lower than that of wild-type, indicating the increased hydrophobic interaction, which could have contributed to the improved thermostability of D9.

**Conclusions:**

These results showed that the thermostability of variant D9 was increased. The variant D9 could be expected to be a promising tool enzyme for its wider industrial applications. The method of truncating the flexible region used in our study has the potential to be used for enhancing the thermostability of other proteins.

**Electronic supplementary material:**

The online version of this article (10.1186/s12934-018-1012-x) contains supplementary material, which is available to authorized users.

## Background

Trypsin (EC 3.4.21.4) is a serine protease, which hydrolyzes peptide bonds on the carboxylic sides of lysine and arginine [1]. It exists widely in nature, and has been discovered in bacteria, fungus and mammals [2]. As an essential tool enzyme, trypsin can be applied in variety of fields including pharmaceutical, leather processing, and food processing, especially in proteomic applications about the use of mass spectrometry (MS) to analyze the peptides obtained [3, 4]. The peptides must be denatured to achieve effective digestion, since the native peptide could not be digested by trypsin. However, the peptides denatured by chemicals would complicate the further analytical steps. The problem could be solved by hydrolyzing peptides under high temperature conditions. Therefore, it is necessary to improve the thermostability of trypsins for satisfying the industrial applications.

In recent years, a number of effective approaches, such as enzyme immobilization, protein engineering, and use of cosolvents, have been successfully utilized to improve the thermostability of trypsin [5–7]. For example, Pazhang et al. reported that polyols (sorbitol and glycerol) stabilized trypsin by decreasing the polypeptide chain fluctuations. Among various methods from improving protein stability, the protein engineering attracted more attention due to requiring changes to the primary structure of the enzyme to confer the desired features [8]. Up to date, protein engineering comprises two main approaches including directed evolution and rational design. The process of directed evolution is heavily experimental and time-consuming. And high-throughput screening is usually required in directed evolution. However, not all enzyme stabilities are amenable to developing a high-throughput screening method. Recently, rational design has been widely applied for engineering the protein thermostability. Compared to directed evolution, rational design is faster and universal because it focuses more on several specific target sites.

With flexible sites as target sites, the strategy of RFS (rigidifying flexible sites) has been a powerful method to engineer the protein thermostability [9]. Identifying the flexible regions was the critical procedure for designing thermostable proteins using this strategy. Molecular dynamics (MD) simulation was commonly applied to identify the highly flexible regions of proteins. However, disadvantages, such as computational expense and inaccuracy, limit the use of MD simulation [10]. Two other methods (FlexPred and FoldUnfold) could also be used to precisely predict the flexible regions, which are faster and cheaper than the MD simulation [11]. When the three methods are used in combination, it has the potential to be more effective to find the highly flexible region.

Rigidifying the flexible regions was another step in the RFS strategy for engineering the protein thermostability [9]. Among various approaches applied to rigidify the flexible regions, the method of truncating flexible regions has received attention in recent years [12–14]. Ban reported that truncation of a flexible portion of the C-terminal end in GBE_*Gt*_ (1,4-α-glucan branching enzyme from *G. thermoglucosidans* STB02) enhanced the thermostability of the enzyme without significantly compromising its enzyme activity [15]. Truncating the flexible N-terminal contributed to increasing thermal stability of mannanase Man 1312 without activity loss [16]. Thus, it is possible to enhance the thermal stability of proteins through truncating the flexible regions.

Ω-loop, a nonregular secondary structure, commonly connects α-helices and β-strands [17]. As a special loop, Ω-loop usually consists of 6–16 amino acids and locates on the surface of proteins, which plays essential roles in protein folding, function and stability [18]. Structural analysis of psychrophilic, mesophilic and thermophilic enzymes revealed that shorter and fewer surface loops are usually existed in thermophilic enzymes due to the higher structural rigidity [19]. Thompson et al. found that the thermostability of proteins could be improved by deleting the dynamic surface loop [20]. Similarly, the thermostability of HCA II could be improved through truncating the surface loop [21]. Therefore, truncation could be an effective way to rigidify the flexible loop and improve the thermostability of trypsin.

**Fig. 1:**
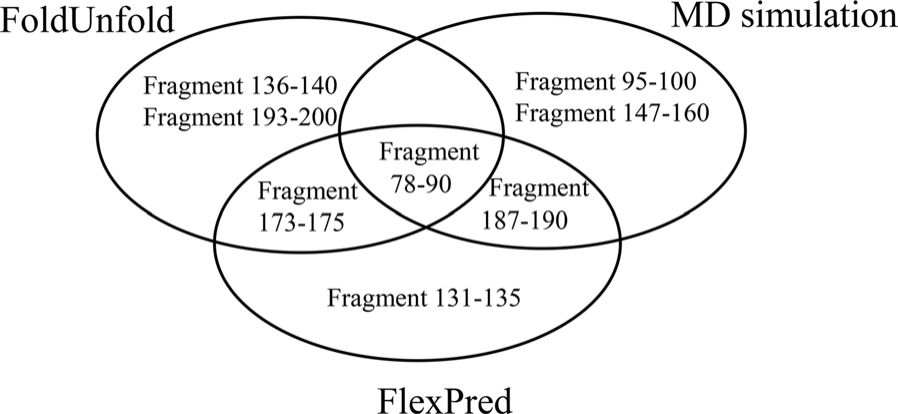
Prediction of the flexible regions of porcine trypsin. MD simulations were carried out in Gromacs package. The RMSF values of WT were collected at 300 K for a minimum of 10 ns. FlexPred predicted residue flexibility in trypsin using SVM approach. The approach of FoldUnfold used the expected average number of contacts per residue calculated from the amino acid sequence as an indicator of whether the given region is folded or unfolded

**Fig. 2:**
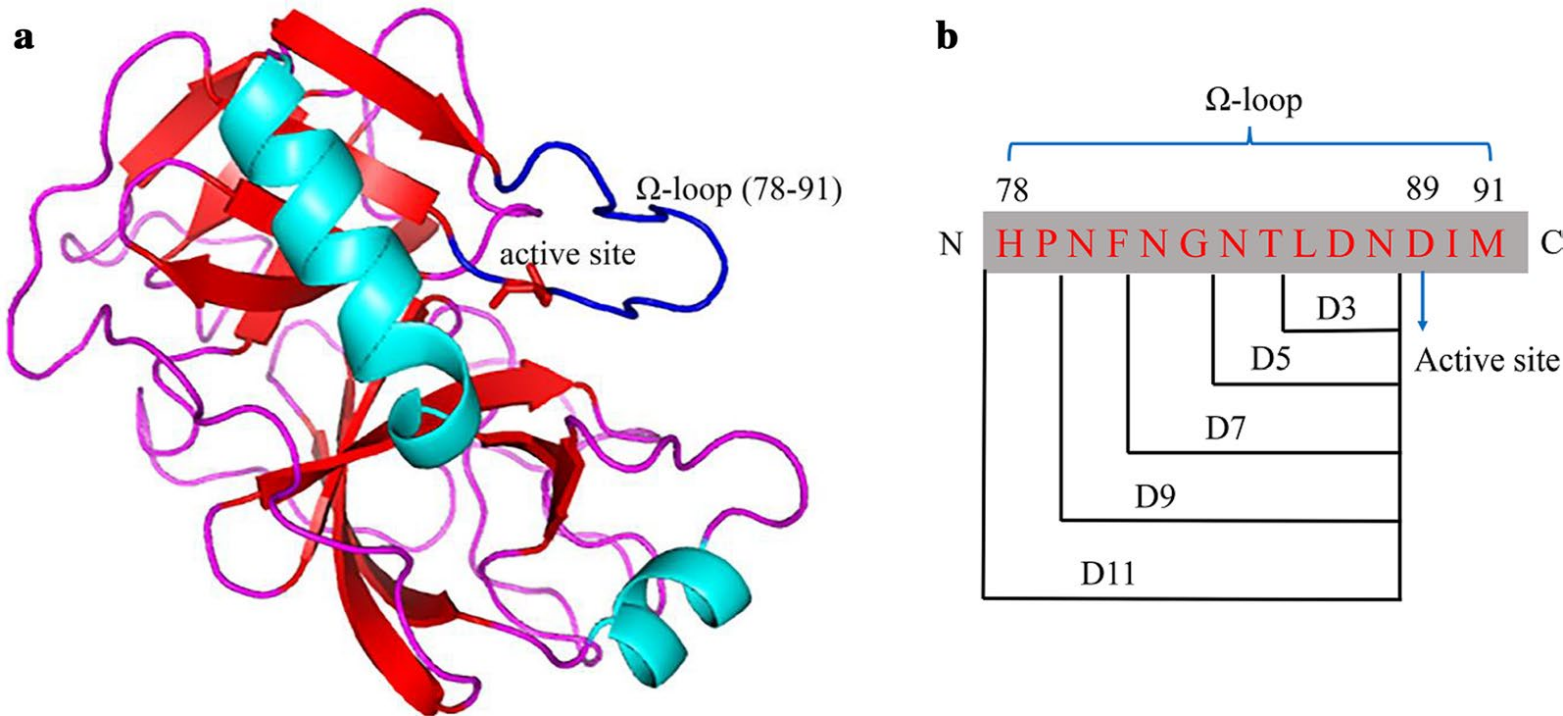
Design of the truncation of the Ω-loop region. **a** Crystal structure of porcine trypsin visualized by the PyMOL software from the Protein Data Bank (PDB ID: 4AN7). The Ω-loop is colored blue and the active site is colored red. **b** The design of the truncation of the Ω-loop region

In this study, we aimed to enhance the thermostability of the trypsin expressed in *Pichia pastoris* GS115 using the strategy of RFS. MD simulation, FlexPred, and FoldUnfold were firstly used to predict the highly flexible regions. It was found that the predicted flexible region (amino acids 78–90) was overlapped with the Ω-loop region (amino acids 78–91) (Figs. 1, 2a). Five variants were constructed by truncating the flexible Ω-loop. The effects of loop truncation on the kinetic properties, thermostability and structural properties were determined. The best variant (D9) showed significantly improved thermal stability without compromising enzymatic activity.

## Results

### Design and purification of the trypsin variants

**Fig. 3:**
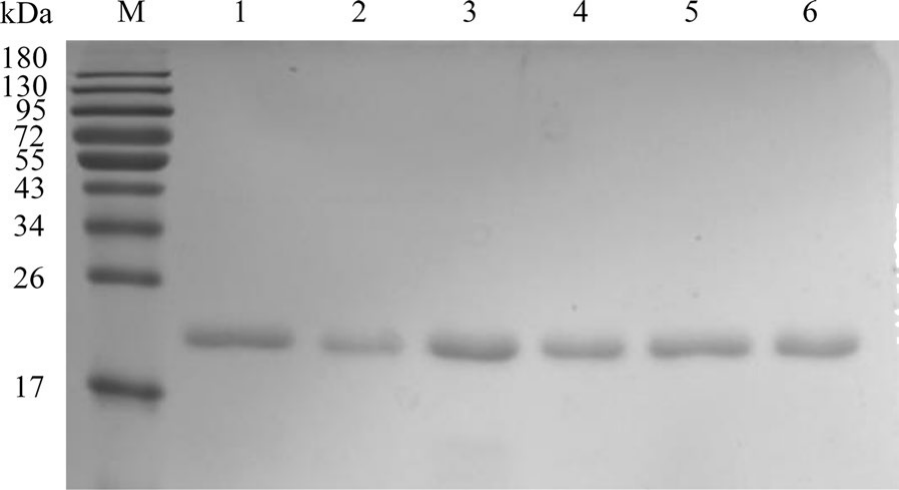
SDS-PAGE analysis for purified WT and variants (D3, D5, D7, D9, and D11) (10 μL; protein concentration 3 μg). M, molecular weight marker; 1, WT; 2–6, D3, D5, D7, D9, and D11

In this study, we attempted to enhance the thermostability of trypsin by the approach of rational design. The flexible regions were predicted by three methods (MD simulation, FoldUnfold, and FlexPred) and listed (Additional file 1: Fig. S1 and Table S1). We selected four flexible regions from each method and compared with each other. The amino acids 78–90 was predicted to be the highly flexible region of the trypsin, which is overlapped with the Ω-loop region (amino acids 78–91) (Figs. 1, 2a). The Ω-loop (amino acids 78–91) was placed between the two short β-strands based on the analysis of three-dimensional porcine trypsin structure, which seemingly interrupts the formation of one long strand. Moreover, based on previous reports, loops were generally considered as potential targets for engineering proteins with improved properties [13, 22–24]. Then five variants (D3, D5, D7, D9, and D11) were designed by truncating the flexible loop by three residues (D3), five residues (D5), seven residues (D7), nine residues (D9) and eleven residues (D11) (Fig. 2b, Additional file 1: Fig. S2). A SP-Sepharose column was used for purifying the variants. The results of SDS-PAGE indicated that all purified enzymes showed a single band of ~ 24 kDa (Fig. 3).

Furthermore, we measured the protein yield based on the purified enzymes. The protein yield of WT was 43.32 mg/L, while the yields of D3, D5, D7, D9, and D11 were 25.82 mg/L, 29.92 mg/L, 53.44 mg/L, 40.58 mg/L, and 33.49 mg/L, respectively. Apparently, the protein yields of other variants were lower than that of WT except for D7. The results suggested that the protein yield might be influenced after truncating the flexible region of trypsin.

### Analysis of thermostability properties

**Fig. 4:**
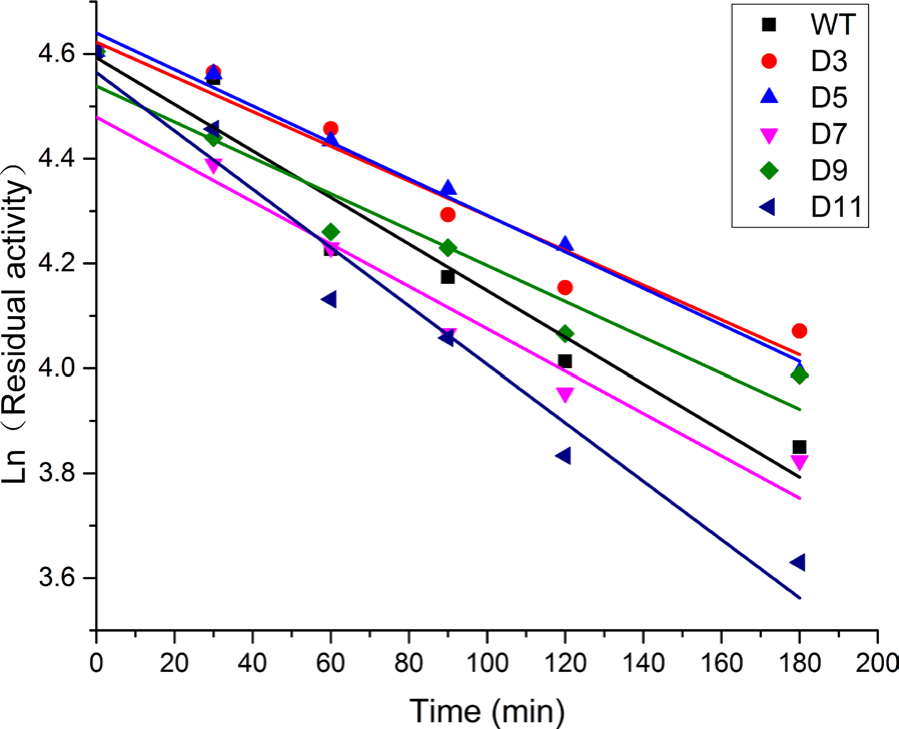
Natural-logarithmic plot of residual activity of WT and variants (D3, D5, D7, D9, and D11). After incubation at 50 °C for different minutes, the residual activity of each trypsin was assayed. The original activity was defined as 100%. Each value is the mean of three independent experiments

The term “protein stability” commonly includes kinetic stability and thermodynamic stability [25]. To investigate kinetic stability of WT and variants, purified enzymes were incubated at 50 °C for different time (30 min, 60 min, 90 min, 120 min, and 180 min, respectively) and then the residual activity was measured at 25 °C. The effect of the heat treatment on enzyme activities was shown in Fig. 4 and Table 1. The *k*_d_ values of variants were lower than that of WT except D11, indicating that WT had deactivated more slowly than D11 and more rapidly than other variants. After incubation at 50 °C for 60 min, WT, D7 and D9 had 70% remaining activities, while the residual activity of D11 was nearly 60% (Additional file 1: Fig. S3). Meanwhile, both variants D3 and D5 retained more than 85% of the original activities. The half-life value of WT was 155.76 min, while the half-life times of D3, D5. D7, D9, and D11 were 209.41 min, 199.18 min, 171.57 min, 202.08 min, and 124.44 min, respectively (Table 1). Except D11, all other variants showed longer half-life time than WT, indicating that the kinetic stability of trypsin was improved by truncating the Ω-loop region.

**Table 1:** Thermostability constants of WT and variants

Enzymes	*T*_opt_ (°C)	*T*_m_ (°C)	$T_{50}^{10}$ (°C)	*t*_1/2_ (50 °C) (min)	*k*_d_ (min^−1^)
WT	60.0	51.0	54.5	155.76	4.45 × 10^−3^
D3	65.0	50.5	65.8	209.41	3.31 × 10^−3^
D5	60.0	62.3	62.0	199.18	3.48 × 10^−3^
D7	55.0	54.4	61.2	171.57	4.04 × 10^−3^
D9	65.0	55.5	60.3	202.08	3.43 × 10^−3^
D11	50.0	53.3	60.5	124.44	5.57 × 10^−3^

Each value was calculated from triplicate experiments

**Fig. 5:**
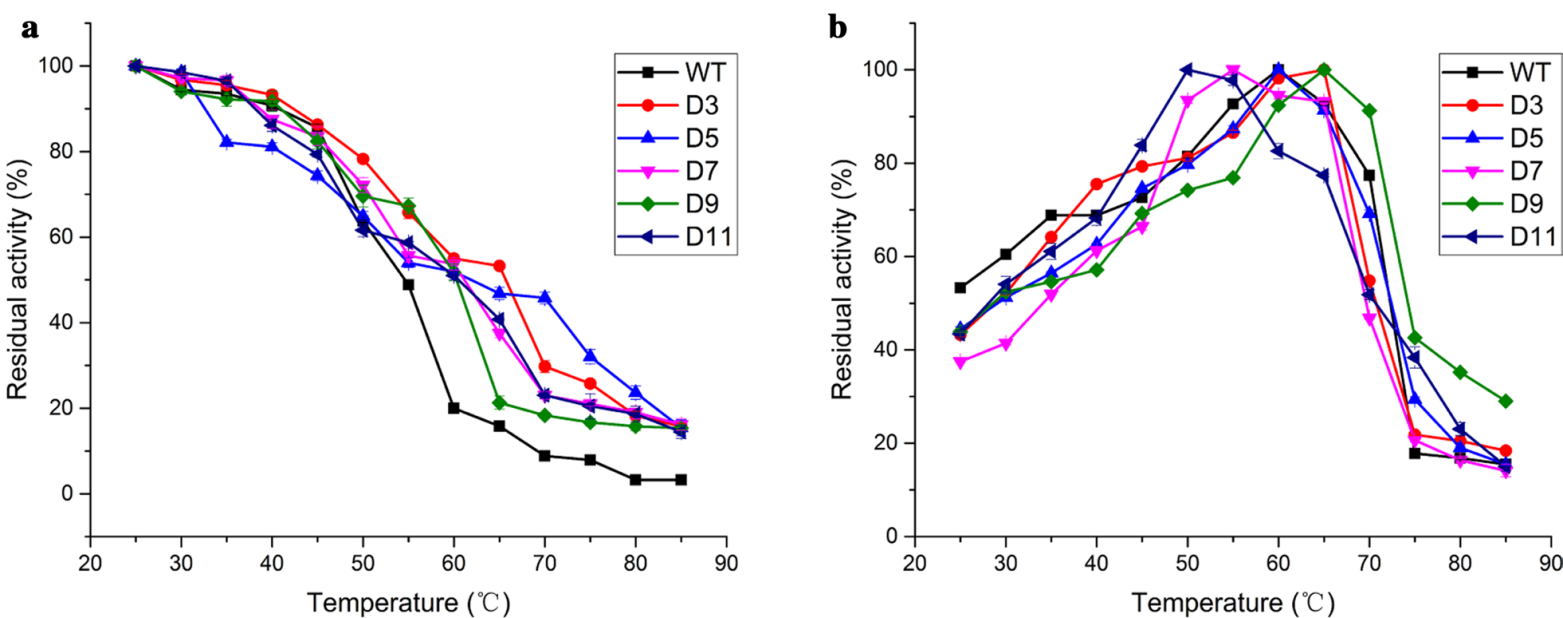
Thermostability properties of WT and variants (D3, D5, D7, D9, and D11). **a** Comparison of thermal tolerance for WT and variants. The residual activities of the enzymes were measured after incubating at different temperatures (25–85 °C) for 10 min. **b** Effect of temperature on enzyme activity. The residual activities were determined under different temperatures (25–85 °C). Values are presented as the average of three experiments

The inactivation curves of WT and variants were obtained by incubating the enzymes at temperatures ranging from 25 to 85 °C for 10 min. After heat treatment, enzymes were cooled in the icebox for 5 min. As shown in Fig. 5a, WT almost lost their enzyme activities at 85 °C, but other variants remained nearly 15% of its original activity. Moreover, the residual activity of all variants at 60 °C was above 50%, while WT only retained 20% of initial activity. The $T_{50}^{10}$ values of D3, D5, D7, D9, and D11 were determined to be 65.8 °C, 62.0 °C, 61.2 °C, 60.3 °C, and 60.5 °C, respectively, while that of WT was 54.5 °C (Table 1).

### Melting temperature measurement

To investigate the effect of truncation on the thermodynamic stability, the thermoflour assay using the SYPRO Orange as a dye was determined. SYPRO Orange is weakly fluorescent in the hydrophilic environment but has a high fluorescence intensity in hydrophobic regions of proteins [26]. Along with the protein unfolding, the dye binds to the exposed hydrophobic regions of the protein which could lead to a significant increase in the fluorescence intension. As shown in Table 1 and Additional file 1: Fig. S4, *T*_m_ value for WT was 51.0 °C, whereas *T*_m_ values of variants were 50.5 °C, 62.3 °C, 54.4 °C, 55.5 °C, and 53.3 °C for D3, D5, D7, D9, and D11, respectively. The thermodynamic stability of variants of D5, D7, D9, and D11 was higher than that of WT, while the thermodynamic stability of D3 was decreased.

### Optimum temperature for WT and variants

The influence of temperature on the enzymatic activity of WT and variants was investigated. The enzyme activity profile was measured between 25 and 85 °C, with 5 °C intervals. As shown in Fig. 5b and Table 1, the optimum temperatures of D3 and D9 were 65 °C, 5 °C higher than that of WT, while the optimum temperatures of D7 and D11 were decreased by 5 °C and 10 °C, respectively. The optimum temperature of D5 was 60 °C, which was consistent with that of WT. At 85 °C, the variant D9 retained nearly 30% of its initial activity, while the residual activity of other variants and WT was about 15%.

### Characterization of kinetic properties

Kinetic parameters of WT and variants were shown in Table 2. The *K*_m_ and *k*_*cat*_ values of WT were 28.80 ± 2.94 μM and 0.634 ± 0.030 s^−1^, respectively. The *K*_m_ values of D7 and D11 were drastically decreased, and that of D3 was increased by more than 50%. Furthermore, the *K*_m_ values of D5 and D9 were 33.40 ± 4.11 μM and 27.04 ± 3.35 μM, respectively. Although *k*_cat_ values displayed no significant difference between WT and variants, the *k*_cat_/*K*_m_ values of D7 and D11 were around 150% and 250% higher than that of WT, respectively. In addition, the *k*_cat_/*K*_m_ value of D3 was decreased by nearly 50%, while other variants showed no considerable change compared with WT. The specific activity of WT was 105,614.2 ± 4154.28 U/mg, while the specific activity of D3, D5, D7, D9, and D11 was 73,850.3 ± 1385.34 U/mg, 102,234.8 ± 5238.41 U/mg, 196,961.5 ± 8182.93 U/mg, 108,760.9 ± 5205.32 U/mg, and 340,425.6 ± 9314.77 U/mg, respectively (Table 2). The specific activity of trypsin displayed the same tendency of changes with the catalytic efficiency after truncating the Ω-loop region.

**Table 2:** Kinetic parameters of WT and variants

Enzymes	*K*_m_ (μM)	*k*_cat_ (s^−1^)	*k*_cat_/*K*_m_ (mM^−1^ s^−1^)	Specific activity (U/mg)
WT	28.80 ± 2.94	0.634 ± 0.030	22.014 ± 1.45	105,614.2 ± 4154.28
D3	58.60 ± 3.30	0.676 ± 0.007	11.536 ± 0.87	73,850.3 ± 1385.34
D5	33.40 ± 4.11	0.652 ± 0.007	19.521 ± 1.05	102,234.8 ± 5238.41
D7	11.01 ± 0.94	0.604 ± 0.024	54.910 ± 3.56	196,961.5 ± 8182.93
D9	27.04 ± 3.35	0.624 ± 0.099	23.111 ± 2.19	108,760.9 ± 5205.32
D11	7.50 ± 0.29	0.580 ± 0.019	77.330 ± 4.98	340,425.6 ± 9314.77

Each value was calculated from triplicate experiments. Means ± standard deviations

The enzyme activities were measured at 25 °C

### CD spectroscopy analysis

**Fig. 6:**
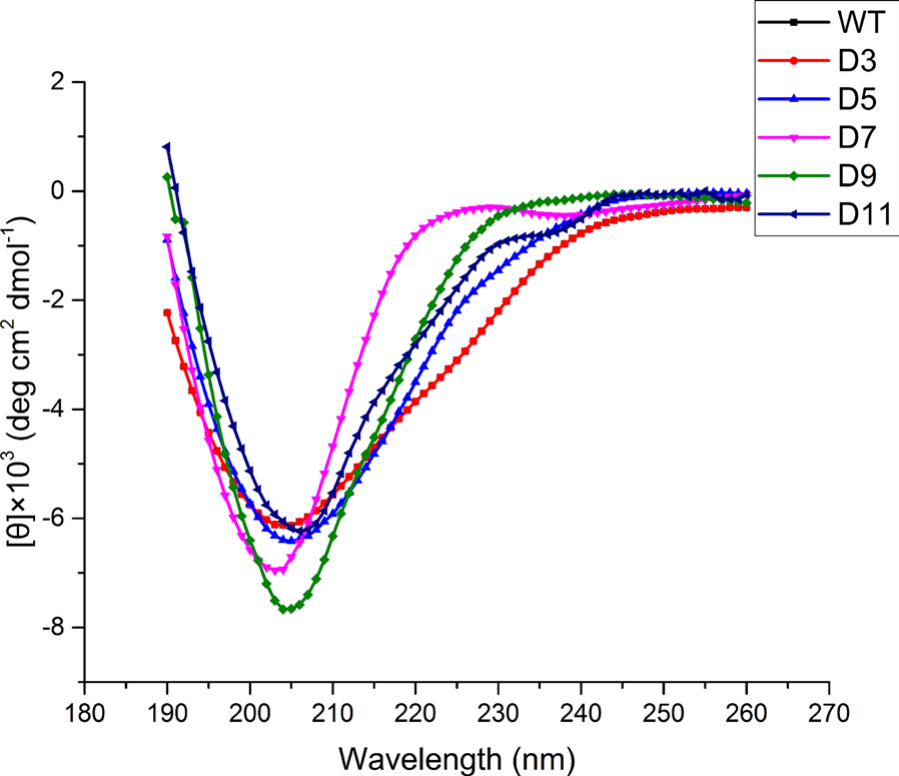
CD spectra of WT and variants (D3, D5, D7, D9, and D11) were measured at room temperature (25 °C). The concentration of protein used for the far-UV CD spectrum (190–260 nm) was 0.2 mg/mL. Each enzyme equilibrated in Tris–HCl buffer (10 mM, pH 8.0)

CD spectra and fluorescence spectra were performed to further explore the impact of the truncation on structures. As shown in Fig. 6, CD spectra of WT and all variants exhibited a similar shape. The spectrum of WT and its variants all showed a single negative band near to 205 nm, indicating that the percentage of the secondary structure had no considerable alteration (Additional file 1: Table S2).

### Fluorescence spectroscopy analysis

**Fig. 7:**
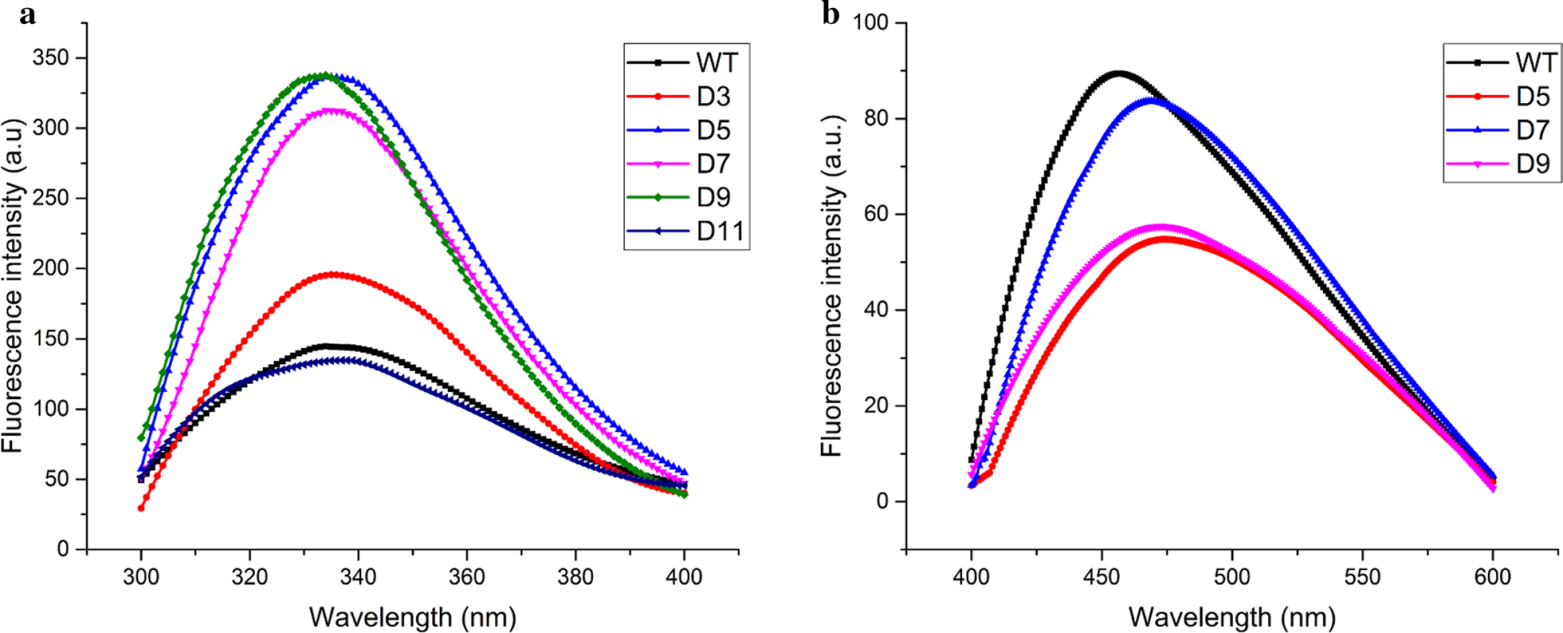
Fluorescence spectra of WT and variants (D3, D5, D7, D9, and D11). **a** Intrinsic fluorescence spectra of WT and variants. The fluorescence emission spectra were monitored at 300–400 nm. **b** Surface hydrophobicity of WT and variants. The fluorescence emission spectra were recorded between 400 and 600 nm

Intrinsic fluorescence experiment was carried out to estimate the influence of mutations on the tertiary structure of trypsin. As shown in Fig. 7a, except for D11, the result of fluorescence spectra displayed that WT and other variants had a maximum emission at around 334 nm. It indicated that there was no significant change in tertiary structures after the truncation the Ω-loop region. However, a redshift of 3 nm was found between WT and D11, which indicated partial tryptophan residues of D11 more exposed to the hydrophilic environment. In addition, the emission intensity of D3, D5, D7, and D9 were notably higher than that of WT, suggesting that the tryptophan residues were buried away from the aqueous solvent.

The surface hydrophobicity of trypsin was determined by fluorescence spectra. As shown in Fig. 7b, the fluorescence intensity of WT was higher than variants (D5, D7, and D9). The results indicated that the three variants had a significant decrease in the surface hydrophobicity compared with WT.

## Discussion

Trypsin is the most widely used protease for the studies about proteins. In many instances, a high thermostability of trypsin is usually required in some industrial and biotechnological applications. Some methods, such as immobilization, the addition of polyol and enzymatic glycation, have been used for improving the thermostability of trypsin [6, 27, 28]. In the present study, we improved the thermostability of trypsin by truncating the flexible region. The highly flexible region (amino acids 78–90) was identified, which is overlapped with the Ω-loop region (amino acids 78–91). We designed five variants (D3, D5, D7, D9, and D11) in this flexible loop. Among these variants, D9 variant showed significantly improved thermostability with no loss of enzyme activity. In addition, the variant D9 showed no significant change in structures when comparing with WT. The properties of D9 suggest that truncating the flexible regions could be an effective strategy for improving the thermostability of proteins.

In this work, MD simulation was used to determine the overall and local flexibility of WT and D9 variant. The overall flexibility of proteins was evaluated using RMSD values. And the local flexibility of proteins was investigated by analyzing the RMSF values. As shown in Additional file 1: Figs. S5, S6, the variant D9 showed lower RMSD and RMSF values compared with WT. From the results, we found that the overall and local flexibility of D9 variant were all decreased, which might be a reason that the thermostability of D9 variant was increased. Previous studies have showed that the proteins with better thermostability which usually displayed higher rigidity [9, 29]. Furthermore, we also predicted the residue fluctuation profile by submitting the protein structures to CABS-flex sever. As shown in Additional file 1: Fig. S7, the Ω-loop located at a region with high mobility, suggesting that these residues in this region underwent large disturbance. And the fluctuations in this region were considerably alleviated when the Ω-loop was truncated. This result might be related to the enhancement of the hydrophobic interaction in variant D9. Hydrophobic interaction plays an essential role in the stability of protein structure [30]. Due to the energetic cost of solvating nonpolar side chains, the exposure of hydrophobic residues at the protein surface may lead to a decrease in protein thermostability [31]. The increase in the hydrophobicity of protein’s internal residues or the decrease in the hydrophobicity of protein’s surface residues improves the hydrophobic interaction of proteins [32, 33]. In this study, the surface hydrophobicity of D9 was lower than that of WT, indicating the increased hydrophobic interaction.

Enhancing stability without sacrificing enzyme activity is critical for protein engineering [34]. The improvement of thermostability generally leads to the decrease of enzyme activity due to the so-called stability-activity trade-off [35]. As shown in Table 2, we observed that the change in enzyme activity was mainly caused by an alteration of the *K*_m_ value but not the *k*_cat_ value. The thermostability of D3 was significantly increased, while the catalytic efficiency of D3 was decreased by nearly 50% compared with WT. The *K*_m_ of D3 was increased by 1.03-fold, indicating that the substrate binding affinity was affected after deleting the three residues at the Ω-loop region. In contrast, the variant D11 has the lowest melting temperature and shortest half-life among these variants. The *K*_m_ of D11 was drastically decreased and its *k*_cat_ value was decreased only slightly, resulting in the highest catalytic efficiency (Table 2). The reason for this might be the change of surface charge between WT and D11 (Additional file 1: Fig. S8). Vidya et al. found that the alteration of surface charge affected the thermal properties of l-asparaginase II [36]. And Niu et al. reported that the variant BglT with high activities displayed the biased distribution of surface charge compared with the wild-type BglT [29]. In this research, we investigated the electrostatic surfaces of WT and D11 using PyMOL. The potential distribution of WT exhibited neutral in the Ω-loop region, while the variant D11 displayed almost all negative in this region. Furthermore, we inferred that the decrease of thermostability of D11 might be related to the loss of aromatic π–π interactions. The aromatic π–π interaction plays an important role in protein stability and the loss of aromatic π–π interaction could decrease the protein stability. For instance, Deng et al. found that aromatic π–π interaction was more powerful than hydrogen bonds and salt bridges in stabilizing α-amylase [37]. Thus, we analyzed aromatic π–π interactions of D11 by PIC server, and it revealed that the variant D11 lacked three aromatic π–π interactions when compared with WT. Nevertheless, the activity-stability trade-off could be overcome through creating chemically and/or genetically modified nonnatural enzymes [19]. In this study, the thermostability and catalytic efficiency of D9 are all increased compared with WT. Based on the crystal structure, we found the active site of Asp89 was located at the Ω-loop region (Fig. 2a). The active site was usually covered by the adjacent loops, which might influence the enzyme activity [38]. Moreover, Zhang et al. reported that rigidifying the flexible residues in the active center improved the thermostability of *Candida rugosa* lipase1, without a decrease in catalytic activity [39]. Thus, truncating the Ω-loop region might result in the improvement of the thermostability and enzyme activity at the same time.

CD spectra and fluorescence spectra were measured to investigate the structural changes between the WT and variants. Compared with WT, the CD spectra of all variants showed no obvious change. It indicated the truncation of the Ω-loop region have almost no influence on the secondary structure of trypsin. Moreover, according to the results of fluorescence experiments, the intrinsic fluorescence intensity of variants (D3, D5, D7, and D9) showed a superior increase compared with WT, suggesting the structure of enzyme became tighter after truncating the Ω-loop region. This result was coincident with the work of Kheirollahi and Khajeh [34]. They found that the higher intrinsic fluorescence intensity might be the result of the increase of structure compactness in the variant Glu138Pro. Moreover, Boone et al. suggested that the thermal stability of HCA II can be increased by improving the surface compactness via loop truncation [21].

Accurate prediction of flexible regions is critical for engineering the thermostability of proteins through the method of RFS. Based on the analysis of MD simulation, the flexible region (amino acids 78–90) is not one of the most flexible regions (Additional file 1: Fig. S1). This means that the flexible region could be eliminated if only predicted by MD simulation. Therefore, it is important to use several predictive methods simultaneously for engineering the protein thermostability.

## Conclusions

In this study, the flexible regions of trypsin were predicted by three methods (MD simulation, FlexPred, and FoldUnfold) and the highly flexible region were identified through the combination of three methods. Based on the above prediction, we modified this region by the method of truncation and acquired five variants. Compared with WT, the D9 variant showed a 5 °C, 5.8 °C, and 4.5 °C increasement in *T*_opt_, $T_{50}^{10}$, and *T*_m_, respectively. The half-life value of D9 was 202.08 min, 46 min longer than that of WT. These results demonstrated that truncating the flexible regions could benefit the improvement of thermostability. In addition, this study provides an efficient method that could be applied to engineer other proteins for better thermostability.

## Methods

### Analysis of flexible regions

MD simulation was measured using the Gromacs package, version 5.0 with the Gromos force field (53A6). The initial structure was solvated in a cubic simulation box with a layer of water at least 1.1 nm from the protein surface. The whole system was minimized using the steepest descent method (1000 steps) in addition to the conjugate gradient method (5000 steps). Then two 100 ps position-restricted simulations were measured under NVT and NPT ensembles, respectively. Finally, a 10 ns MD simulation was performed at 300 K. The values of RMSD (root mean square deviation) and RMSF (root mean square fluctuation) were calculated using g_rmsd and g_rmsf, respectively.

Similarly, the FoldUnfold and FlexPred were also used to predict the flexible regions. The FlexPred web server analyzes the flexibility of proteins using support vector machine (SVM). Moreover, the three-dimensional structure was used as an input for the two methods to predict flexible regions of proteins. The crystal structure of porcine trypsin (PDB ID: 4AN7) [40] was acquired from the Protein Data Bank and the structures of variants were constructed by homology modeling in the SWISS-MODEL web server (http://www.swissmodel.expasy.org) [41]. The residue fluctuation profile of proteins was analyzed by CABS-flex server [42]. Visualization and analysis of structures were performed by PyMOL 2.7.6.

### Strains, plasmids, and chemicals

*Escherichia coli* Top 10 (TransGen, Beijing, China) was used as the host strain for cloning. *Pichia pastoris* GS115 and the pPIC9K vector were from our laboratory collection. Pfu DNA polymerase and restriction endonucleases were purchased from Thermo Fisher Scientific (Waltham, USA). Bradford Protein Assay Kit (TIANGEN, Beijing, China) was used to determine the concentration of proteins. All other chemicals and reagents were analytical grade.

### Recombinant plasmid construction

The gene sequence of the porcine trypsinogen (GenBank Accession No. CS583166.1) was synthesized (GENEWIZ Inc., Tianjin, China) and inserted into the vector pPIC9K to construct the plasmid pPIC9K-Try. Five variants (residues^86–88^/D3, residues^84–88^/D5, residues^82–88^/D7, residues^80–88^/D9, and residues^78–88^/D11) were generated by deleting three amino acids, five amino acids, seven amino acids, nine amino acids and eleven amino acids, respectively, from the N-terminal of Asp89 at the Ω-loop region. The gene sequences of variants used in this study were achieved by overlapping extension PCR. The amplified PCR products and the pPIC9 K vector were digested with *Eco*RI and *Not*I, ligated, and then transformed into *E. coli* Top 10. The recombinant plasmids were confirmed by DNA sequencing (GENEWIZ Inc., Tianjin, China), and then transformed in *P. pastoris* GS115. All the primers used to acquire the variants in this study were shown in Additional file 1: Table S3.

### Expression and purification of trypsin variants

The positive clones of the wild-type (WT) and variants were inoculated into shake flask containing 25 mL BMGY (buffered glycerol-complex) media and cultured at 30 °C with 250 rpm shaking speed until the OD reached 2–6. Then centrifuged at 3000×*g* for 5 min to collect the cells and transferred into a 500 mL conical flask contained 100 mL of BMMY (buffered methanol-complex) medium to induce protein expression for 96 h. As an inducer, methanol was added to a final concentration of 1% (v:v) every 24 h. The crude enzyme solutions were purified by a SP-Sepharose column (Bona INC., Jinan, China). Moreover, the protein was eluted using a 0–500 mM sodium chloride gradient. The purification of WT and variants was performed by AKTA system (GE Healthcare, Sweden). The final concentration of enzymes was measured through Bradford method, and 15% SDS-PAGE was used to certify the eluted protein [43].

### Enzyme activity assays

The activity of trypsin was determined using Nabenzoyl l-arginine ethyl ester hydrochloride (BAEE) as a substrate, as described previously [44]. Initially, the assay was the trypsinogen activated by enterokinase. Furthermore, BAEE was hydrolyzed by trypsin to Nabenzoyl l-arginine (BA). Trypsin reaction was conducted in a 3 mL of assay buffer (67 mM NaH_2_PO_4_ buffer, containing 0.25 mM BAEE, pH 7.6). The change in absorbance at 253 nm was measured in a UV-765 PC spectrophotometer (Youke Co., Ltd, Shanghai, China). One unit (U) was defined as enzyme required for producing an ∆OD_253_ of 0.001 per minute at 25 °C.

### Measurement of thermostability

Digestion with trypsin is usually carried out at 20–40 °C [27]. It provides the possibility of carrying out digestions at higher temperatures if the porcine trypsin was found to be remarkably stable at 50 °C. Thus, for the thermal stability assay, the purified trypsin (200 μg/mL protein in 67 mM NaH_2_PO_4_ buffer, pH 7.6) were heat-treated at 50 °C for 30, 60, 90,120, and 180 min, and then cooled on ice for 30 s. The enzyme activities were measured at 25 °C, and the residual activity was recorded as the percentage of its initial activity. The first-order rate constant (*k*_d_) was obtained by linear regression of ln (residual activity) versus the duration time. The half-time of WT and variants at 50 °C were calculated by the equation. All experiments data were duplicated three times.

The protein half-inactivation temperature ($T_{50}^{10}$) was determined by measuring residual activity at different temperatures. The enzyme solutions were incubated at the temperature range of 25–85 °C for 10 min and then cooled on ice for 5 min. Finally, the residual activity was determined at 25 °C as described above. The value of $T_{50}^{10}$ was defined as the temperature when the enzyme lost half of its activity after the above treatment. The experiments were repeated three times.

### Thermoflour assay

Melting temperatures of WT and variants were determined with the Thermoflour method [26, 45]. Samples containing 2.0 μM protein and 5 × SYPRO Orange (diluted from a 5000 × stock supplied in DMSO) were prepared in sample buffer (50 mM HEPES, 0.5 mM TCEP and 67 mM NaH_2_PO_4_ buffer, pH 7.6). Denaturation curves were determined in a LightCyder 480 Real-time PCR machine (Roche, Mannheim, Germany). The temperature was ranged from 25 to 95 °C at intervals of 0.5 °C. The wavelengths for excitation and emission were 465 and 580 nm, respectively. All determinations were carried out in triplicate. The data were analyzed using a plug-in named LightCycler Thermal Shift Analysis (Roche, Mannheim, Germany) and melting temperature (*T*_m_) was acquired by taking a negative first derivative plot.

### Measurement of optimum temperature

The optimal temperature was assayed at various temperatures ranging from 25 to 85 °C at intervals of 5 °C. For WT and variants, the optimal activity assayed was considered as 100%.

### Determination of kinetic parameters

Kinetic parameters (*K*_m_ and *k*_cat_) were measured at various concentrations of substrate BAEE in 67 mM NaH_2_PO_4_ buffer (pH 7.6). The kinetic parameters were calculated by linear regression analysis of Lineweaver–Burk double-reciprocal plot. The measurements were performed by three times. All of measurements were performed by three times.

### Circular dichroism analysis

Circular dichroism (CD) experiment was conducted on a J-810 spectrometer (Jasco, Japan). Samples (0.2 mg/mL) in 10 mM Tris–HCl buffer (pH 8.0) were added to a 1 mm path length quartz cuvette for CD measurement. Spectra were collected with a scan speed of 200 nm/min and a response time of 1 s. The spectra data were monitored from 190 to 260 nm and were averages of three scans. The secondary structures of WT and variants were analyzed by the CDPro software (http://sites.bmb.colostate.edu/sceeram/CDPro/).

### Fluorescence spectroscopy analysis

The intrinsic fluorescence spectra of trypsin were detected on a Hitachi F-2500 Fluorescence Spectrophotometer (Hitachi, Japan) at a concentration of 10 μg/mL. Samples were excited at 280 nm, and the emission was recorded ranging from 300 to 400 nm. The slit widths were 10 nm. All experiments were carried out at room temperature (25 °C), while all required background corrections were made.

### Surface hydrophobicity analysis

Protein surface hydrophobicity was measured using an 8-Anilino Naphthalene Sulfonic acid (ANS) fluorescent probe reported previously [46, 47]. The concentration of protein samples and the fluorescent probe ANS were 2 μM and 30 μM, respectively. The excitation wavelength was 380 nm, and the ANS emission spectra was monitored between 400 and 600 nm. Measurements were determined by triplicates.

## Additional file

**Additional file 1.** This file includes: **Fig. S1.** RMSF values for WT. **Fig. S2.** Truncation of the flexible region. **Fig. S3.** The thermostability assays of WT and variants (D3, D5, D7, D9, and D11). **Fig. S4.** Melting temperature of WT and variants were detected by thermofluor shift assay. **Fig. S5.** Comparison of RMSD values of WT and D9 variant trypsin calculated from 5 ns MD simulation at 300 K. **Fig. S6.** The residual flexibility of WT and D9 variant trypsin during a 10 ns MD simulation. **Fig. S7.** The analysis of residue fluctuation profile of trypsin. **Fig. S8.** Comparison of the electrostatic surface charge of WT and D11. **Table S1.** The prediction of flexible regions by three methods. **Table S2.** The percentage of secondary structures of WT and variants. **Table S3.** Primers used in this study.

## Abbreviations

RFS: rigidifying flexible sites
MD: molecular dynamic
RMSD: root mean square deviation
RMSF: root mean square fluctuation
SVM: support vector machine
BMGY: buffered glycerol-complex
BMMY: buffered methanol-complex
BAEE: Nabenzoyl l-arginine ethyl ester hydrochloride
TCEP: Tris(2-carboxyethyl)phosphine
ANS: 8-Anilino Naphthalene Sulfonic acid
