# Supplementary material for: Enhanced trypsin thermostability in Pichia pastoris through truncating the flexible region
Source: Microb Cell Fact. 2018 Oct 25;17:165. doi: 10.1186/s12934-018-1012-x (PMC6201580; doi:10.1186/s12934-018-1012-x)
Supplement: Supplementary file 1 — Additional file 1. This file includes: Fig. S1. RMSF values for WT. Fig. S2. Truncation of the flexible region. Fig. S3. The thermostability assays of WT and variants (D3, D5, D7, D9, and D11). Fig. S4. Melting temperature of WT and variants were detected by thermofluor shift assay. Fig. S5. Comparison of RMSD values of WT and D9 variant trypsin calculated from 5 ns MD simulation at 300 K. Fig. S6. The residual flexibility of WT and D9 variant trypsin during a 10 ns MD simulation. Fig. S7. The analysis of residue fluctuation profile of trypsin. Fig. S8. Comparison of the electrostatic surface charge of WT and D11. Table S1. The prediction of flexible regions by three methods. Table S2. The percentage of secondary structures of WT and variants. Table S3. Primers used in this study. [file 12934_2018_1012_MOESM1_ESM.pdf]

Additional Material

Enhanced trypsin thermostability in *Pichia pastoris* through truncating the flexible region

Lin Liu[1,2,3], Haoran Yu[1,2,3,4], Kun Du[1,2,3], Zhiyan Wang[1,2,3], Yiru Gan[1,2,3], and He Huang[1,2,3*]

[1]Department of Biochemical Engineering, School of Chemical Engineering and Technology, Tianjin University, Tianjin 300350, China

[2]Key Laboratory of System Bioengineering, Ministry of Education, Tianjin University, Tianjin 300350, China

[3]Collaborative Innovation Center of Chemical Science and Engineering, Tianjin 300350, China

[4]Present address: Department of Biochemical Engineering, University College London, Gordon Street, London, WC1H 0AH, United Kingdom

* To whom all correspondence should be addressed:

Dr. He Huang

Tel: 0086-22-2740-3389; Fax: 0086-22-27403389;

Email: huang@tju.edu.cn

Figures

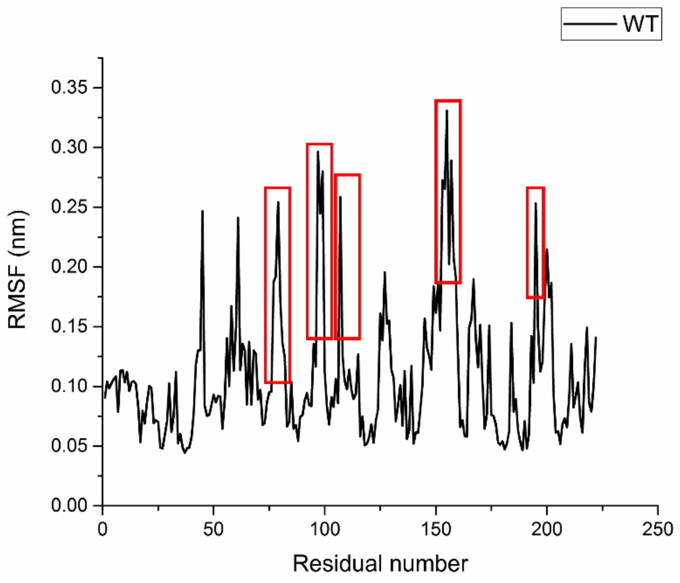

Fig. S1 RMSF values for WT. RMSF of trypsin at 300K versus residue number calculated from 10 ns MD simulation. The flexible regions were shown in the red box.

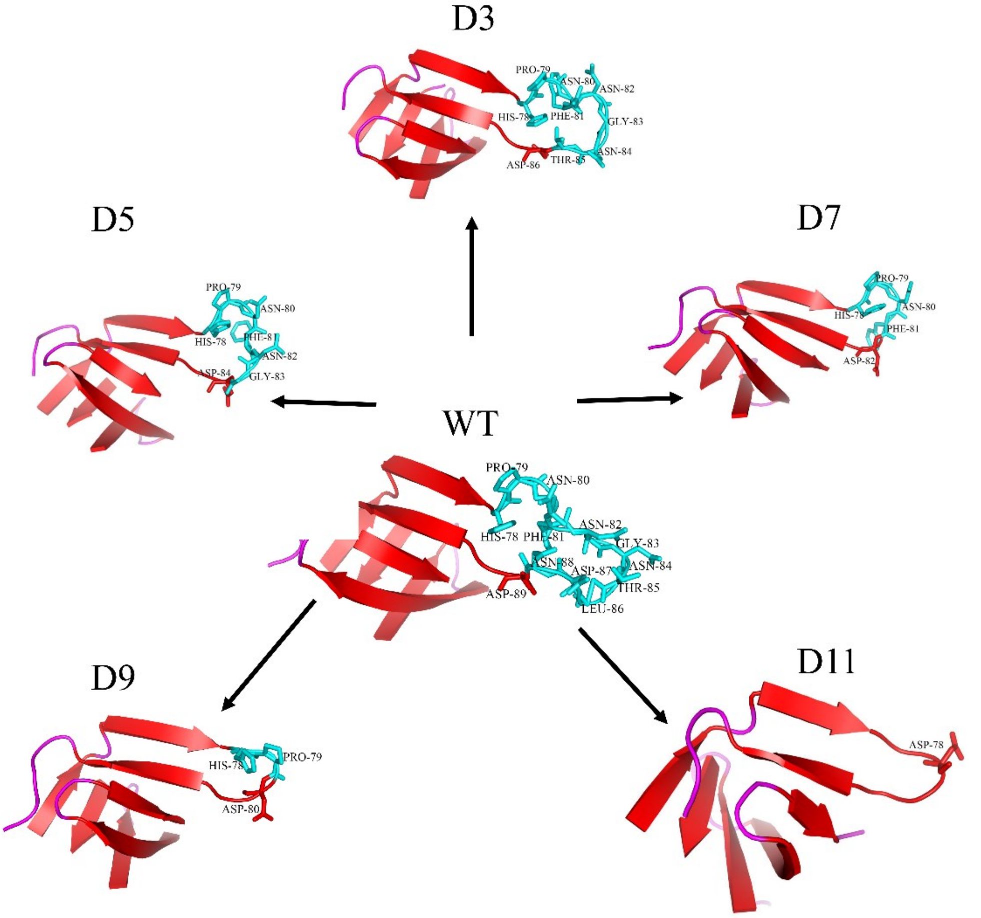

Fig. S2 Truncation of the flexible region. The removal of residues 86-88, residues 84-88, residues 82-88, residues 80-88 and residues 78-88 from the WT, resulting in the variants D3, D5, D7, D9, and D11, respectively.

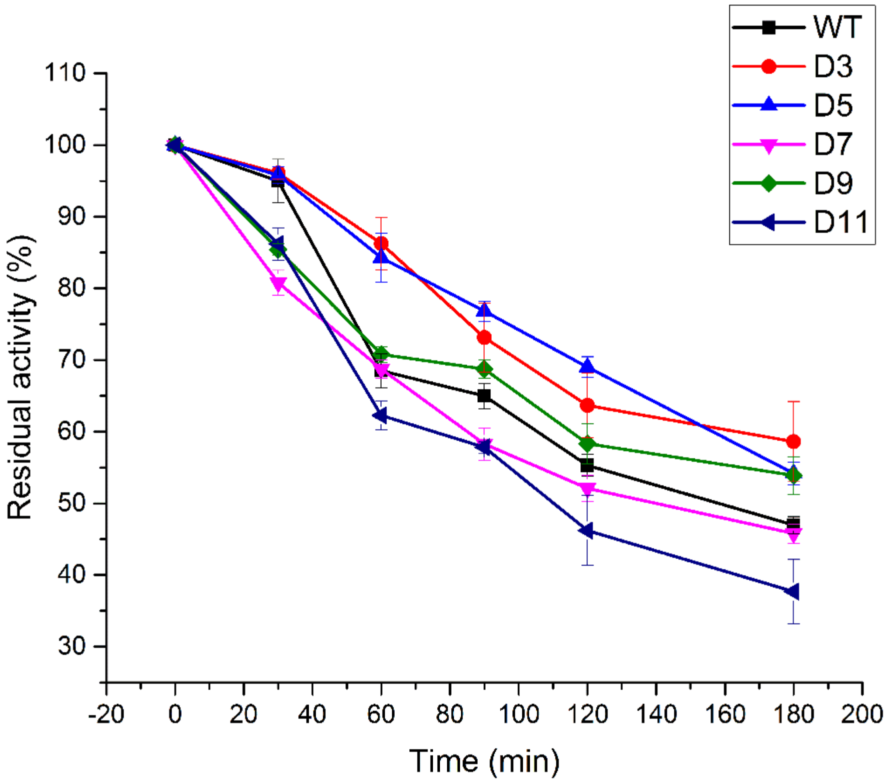

Fig. S3 The thermostability assays of WT and variants (D3, D5, D7, D9, and D11). After incubation at 50 °C for different minutes, the residual activities were measured. Values are mean ± SD (n=3).

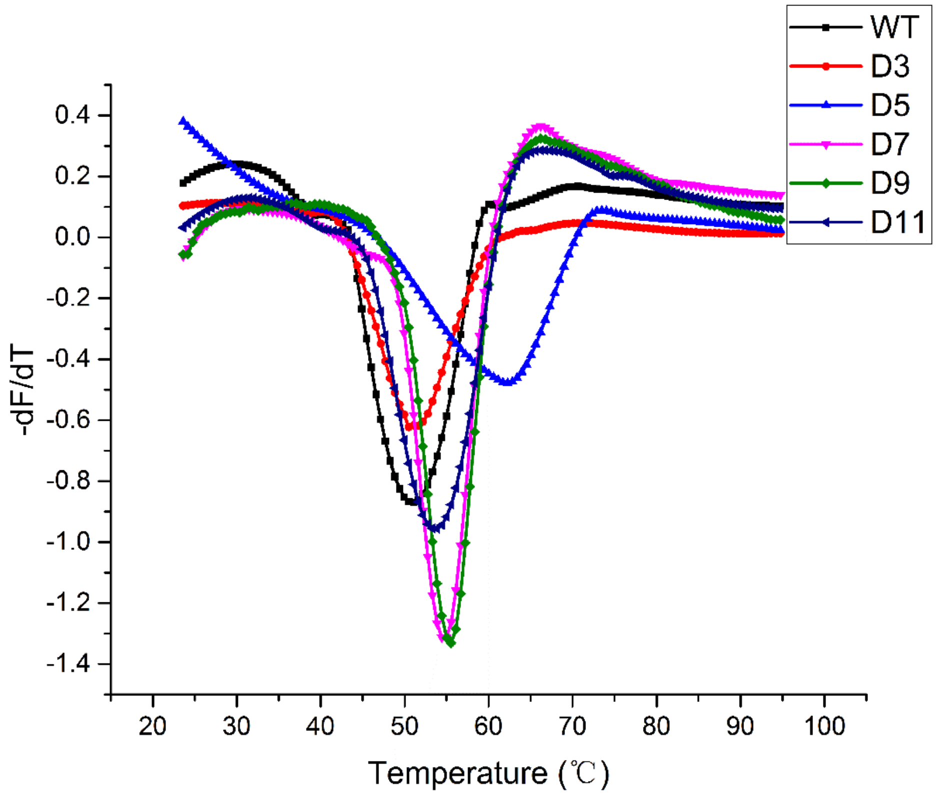

Fig. S4 Melting temperature of WT and variants were detected by thermofluor shift assay.

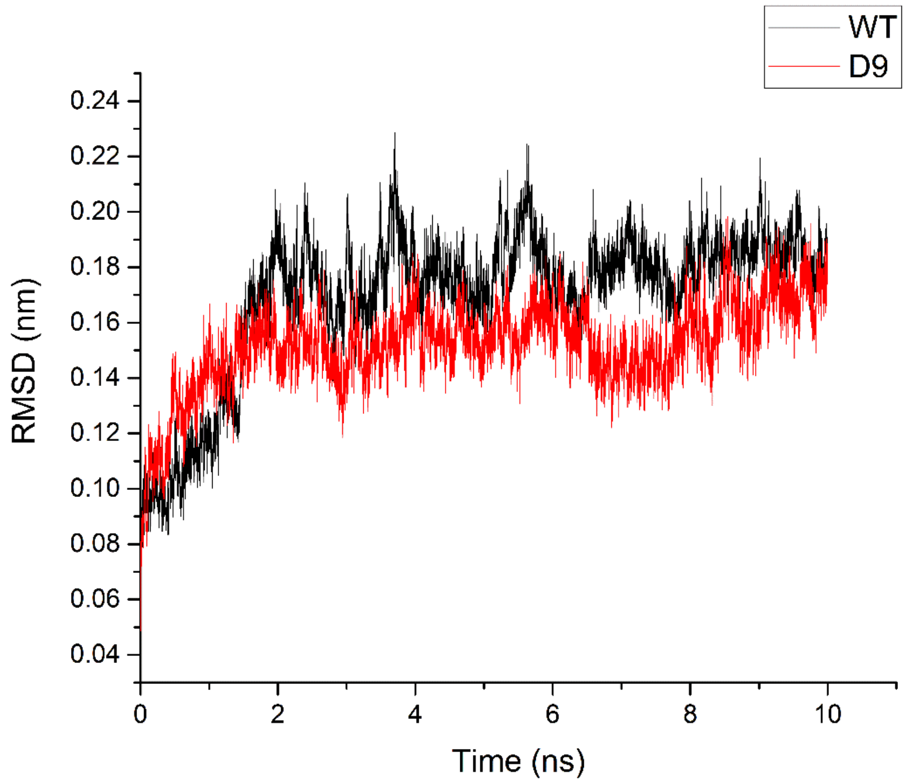

Fig. S5 Comparison of RMSD values of WT and D9 variant trypsin calculated from 5 ns MD simulation at 300 K

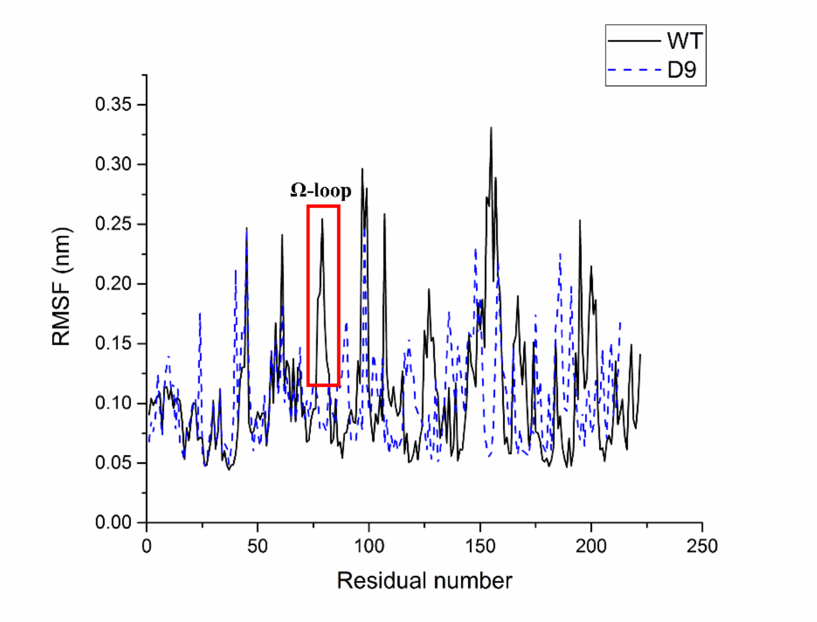

Fig. S6 The residual flexibility of WT and D9 variant trypsin during a 10 ns MD simulation.

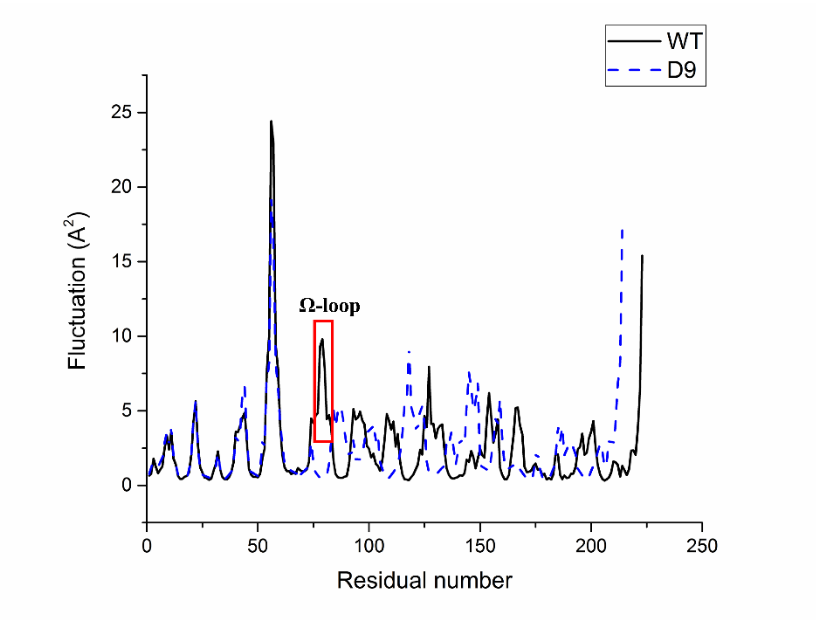

Fig. S7 The analysis of residue fluctuation profile of trypsin. Comparative analysis of residue fluctuation profile between WT and D9 variant. The plot was created by CABS-flex Server. The height at each residue position indicated the average fluctuation level of all atoms in the residue. The fluctuation profile of WT and variant were indicated by black and blue line, respectively.

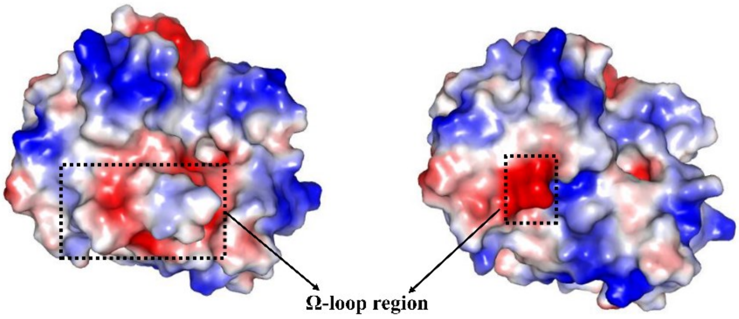

Fig. S8 Comparison of the electrostatic surface charge of WT and D11. Blue, negative charge; red, positive charge.

Tables

Table S1 The prediction of flexible regions by three methods

Methods	Flexible regions
MD simulation	Amino acids 78-91, 106-111, 95-100, 147-160, 187-192
FlexPred	Amino acids 49-56, 76-90, 116-118, 131-135, 173-175, 187-190, 192-198
FoldUnfold	Amino acids 34-39, 78-95, 98-107, 136-140, 150-154, 168-180, 193-200

The flexible regions which were selected from each method were underlined.

Table S2 The percentage of secondary structures of WT and variants

Enzymes	Helix (%)	Strand (%)	Turns (%)	Unordered (%)
WT	21.8	27.0	14.9	35.9
D3	21.6	31.5	12.6	34.4
D5	21.8	31.4	11.4	35.0
D7	21.6	31.9	15.2	30.7
D9	21.9	29.8	14.0	34.2
D11	22.1	29.2	13.5	35.4

Table S3 Primers used in this study

Primer name	Sequence (5'- 3')
PrimerF1-D3	TGGTTCCAATTGACAAGC
PrimerR1-D3	AATTTAATCAACATAATATCAGTATTACCATTAAAATTTGGAT
PrimerF2-D3	GATATTATGTTGATTAAATTGTCTT
PrimerR2-D3	CATTCTGACATCCTCTTGATT
PrimerF1-D5	TGGTTCCAATTGACAAGC
PrimerR1-D5	AATTTAATCAACATAATATCACCATTAAAATTTGGATGAGT
PrimerF2-D5	GATATTATGTTGATTAAATTGTCTT
PrimerR2-D5	CATTCTGACATCCTCTTGATT
PrimerF1-D7	TGGTTCCAATTGACAAGC
PrimerR1-D7	AATTTAATCAACATAATATCAAAATTTGGATGAGTAATAATTT
PrimerF2-D7	GATATTATGTTGATTAAATTGTCTT
PrimerR2-D7	CATTCTGACATCCTCTTGATT
PrimerF1-D9	TGGTTCCAATTGACAAGC
PrimerR1-D9	AATTTAATCAACATAATATCTGGATGAGTAATAATTTTAGCA
PrimerF2-D9	GATATTATGTTGATTAAATTGTCTT
PrimerR2-D9	CATTCTGACATCCTCTTGATT
PrimerF1-D11	TGGTTCCAATTGACAAGC
PrimerR1-D11	AATTTAATCAACATAATATCAGTAATAATTTTAGCAGCATTAAT
PrimerF2-D11	GATATTATGTTGATTAAATTGTCTT
PrimerR2-D11	CATTCTGACATCCTCTTGATT
